# Operationalizing One Health: Environmental Solutions for Pandemic Prevention

**DOI:** 10.1007/s10393-023-01644-9

**Published:** 2023-07-21

**Authors:** Hernan Caceres-Escobar, Luigi Maiorano, Carlo Rondinini, Marta Cimatti, Serge Morand, Carlos Zambrana-Torrelio, Marisa Peyre, Benjamin Roche, Moreno Di Marco

**Affiliations:** 1 Department of Biology and Biotechnologies ‘Charles Darwin’, Sapienza University of Rome, Viale Dell’Università 32, 00185 Rome, Italy; 2 Facultad de Medicina Veterinaria y Agronomía, Universidad de Las Américas, Avenida Manuel Montt 948, edificio A, piso 2, Santiago, Providencia Chile; 3 IUCN Species Survival Commission, Caracas, Venezuela; 4 UMR MIVEGEC, CNRS – IRD, Montpellier University, Montpellier, France; 5 Faculty of Tropical Medicine, Mahidol University, Bangkok, 10400 Thailand; 6 Faculty of Veterinary Technology, Kasetsart University, Bangkok, 10400 Thailand; 7 Department of Environmental Science and Policy, George Mason University, Fairfax, VA USA; 8 CIRAD, UMR ASTRE, Montpellier, France; 9 ASTRE, CIRAD, INRAE, University of Montpellier, Montpellier, France

**Keywords:** Biodiversity, Emerging infectious diseases, Global health security agenda, One health, Pandemic risk, Sustainable development goals

## Abstract

Human pressure on the environment is increasing the frequency, diversity, and spatial extent of disease outbreaks. Despite international recognition, the interconnection between the health of the environment, animals, and humans has been historically overlooked. Past and current initiatives have often neglected prevention under the One Health preparedness cycle, largely focusing on post-spillover stages. We argue that pandemic prevention initiatives have yet to produce actionable targets and indicators, connected to overarching goals, like it has been done for biodiversity loss and climate change. We show how the Driver-Pressure-State-Impact-Response framework, already employed by the Convention on Biological Diversity, can be repurposed to operationalize pandemic prevention. Global responses for pandemic prevention should strive for complementarity and synergies among initiatives, better articulating prevention under One Health. Without agreed-upon goals underpinning specific targets and interventions, current global efforts are unlikely to function at the speed and scale necessary to decrease the risk of disease outbreaks that might lead to pandemics. Threats to the environment are not always abatable, but decreasing the likelihood that environmental pressure leads to pandemics, and developing strategies to mitigate these impacts, are both attainable goals.

## Introduction

The COVID-19 pandemic is the latest novel human infectious disease (NHID) with zoonotic origins and will likely not be the last. While the world’s nations and intergovernmental organizations discuss how to respond to (and recover from) events like COVID-19, an important question remains unanswered: will current responses and recovery strategies reduce the risk of another pandemic?

The emergence of NHID is rooted in the same human activities leading to the current biodiversity and climatic crises, which facilitate conditions for spillover events (Lawler 2009; IPBES 2020; Morand 2020; Lajaunie and Morand 2021; Stephens et al. 2021; Bernstein et al. 2022; Kock and Caceres-Escobar 2022). Despite promising discussions in high-level international fora, the interconnection between the health of the environment, animals and humans has been historically overlooked, as demonstrated by low investment towards environmental solutions (Dobson et al. 2020; IPBES 2020).

The One Health approach is now widely accepted as the best strategy to decrease the risk of future pandemic events, and there are growing international efforts to implement it worldwide (IPBES 2020; Peyre et al. 2021; Morand and Lajaunie 2021a; Adisasmito et al. 2022). One Health is a conceptual framework that recognizes the interconnected and interdependent nature of health among animals, plants, humans, and the environment (Adisasmito et al. 2022). However, past initiatives have fell short of articulating prevention in the One Health preparedness cycle, largely focusing on the post-spillover stages: detection, response, and recovery (Dobson et al. 2020; Bernstein et al. 2022; Johnson et al. 2022). Strategic responses for pandemic prevention should strive for complementarity and synergies among existing initiatives, better articulating prevention as part of the One Health preparedness cycle. Ignoring the interconnections among current planetary crises—increasing health risks, biodiversity loss, climate change—will jeopardize nations’ ability to achieve international commitments, such as the Sustainable Development Goals and the Global Health Security Agenda (Di Marco et al. 2020). Targeting the sources of NHID can potentially control multiple potential threats—known and unknown—rather than targeting single threats.

Global investment on disease preparedness grew in response to COVID-19 (European Commission Directorate-General for Budget 2021), but the consequences of COVID-19 and the acceleration of disease emergence, as well as ecosystem degradation and climate change (McKay et al. 2022), will cast challenges for the years to come. Prevention of pandemic risk is more effective than recovery measures, but a path to operationalize prevention is necessary. Recognizing the importance of making the One Health approach operative, the Quadripartite has recently developed a One Health Joint Plan of Action which defines a set of activities for the period 2022–2026 (FAO et al. 2022). This is an important coordinated effort which strives to identify the overarching steps to implement a global One Health strategy. Yet, we argue that pandemic risk prevention still needs precise targets and indicators aimed at the environmental sources that facilitate spillover and health emergency events. We believe that existing efforts to avert other planetary crises, such as the Convention on Biological Diversity, can provide a pathway to operationalize pandemic prevention at the source.

## Operationalizing Pandemic Prevention Through Environmental Solutions

The One Health Joint Plan of Action defines a vision to achieve “*A world better able to prevent, predict, detect and respond to health threats and improve the health of humans, animals, plants and the environment…*”; the Plan is articulated in a set of expected outcomes, actions, and deliverables. Yet, specific targets, indicators, and baselines are still missing to design, compare and appraise competing interventions to strategically reduce pandemic risk (Baker et al. 2022). These targets and baselines instead exist in other fields such as climate change mitigation (e.g. maintaining the temperature 1.5 °C above pre-industrial levels by 2100) and biodiversity conservation (bringing extinction rates back to pre-Anthropocene levels; Rounsevell et al. 2020). Well-designed targets have the potential to promote collaboration, trust and long-term commitment, while facilitating translating international targets into nationally implemented actions (Maxwell et al. 2015). Likewise, well-designed indicators can effectively monitor the advancement towards achieving the defined targets (Di Marco et al. 2020), identifying state variations of the system, the available data, and the economic cost of collecting new data. There is already a broad understanding on the applications and benefits of indicators to assess the status of species, habitats, and the interacting ecological factors. In fact, several environmental and biodiversity indicators, proposed or already in use by the Convention on Biological Diversity, are potentially relevant for monitoring NHID risk. Importantly, many of these indicators are already reflected in the UN 2030 agenda.

**Figure 1:**
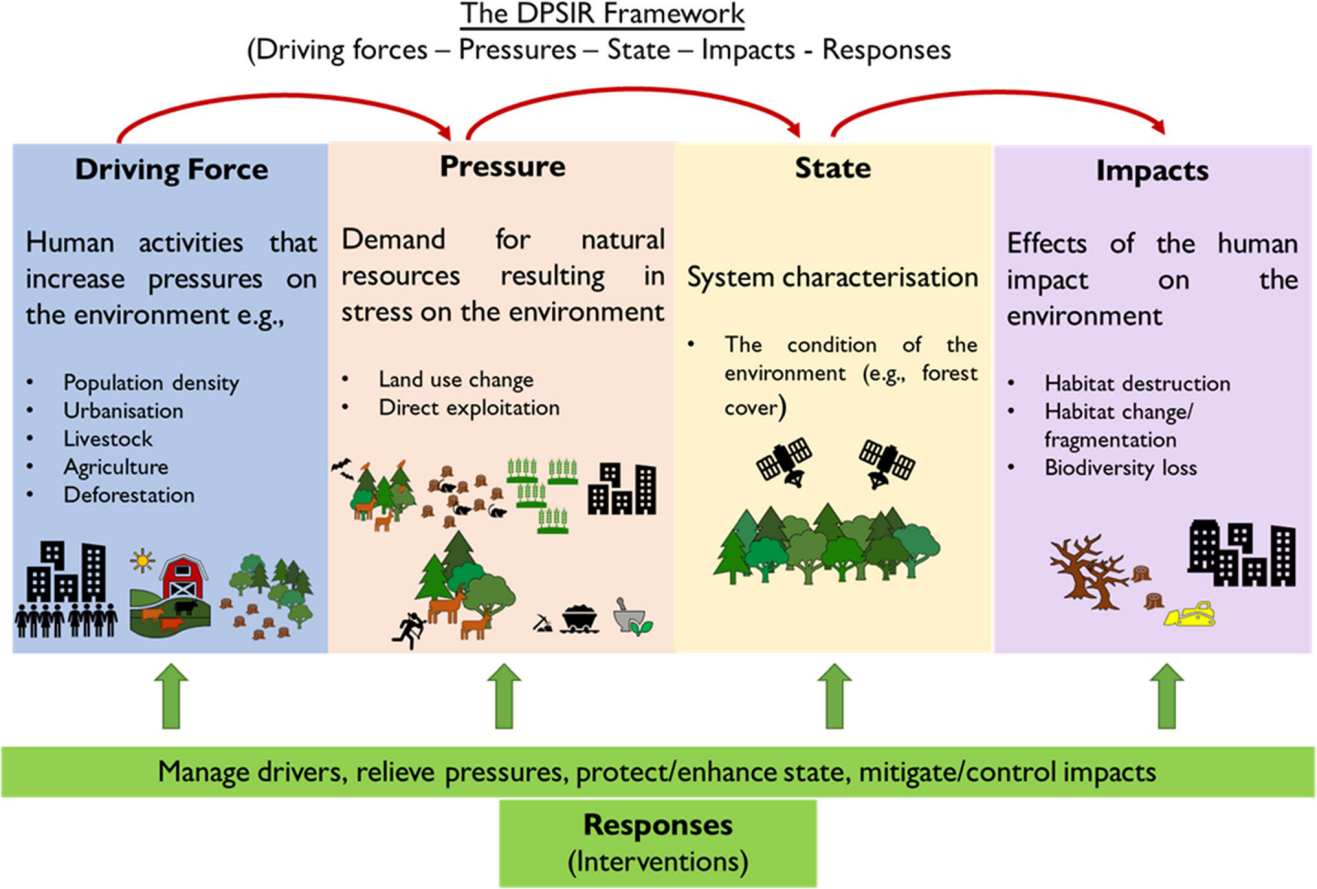
Operationalizing the DPSIR framework for pandemic prevention. The Driver-Pressure-State-Impact-Response framework (or DPSIR). The effects of human activities (Driving Force) increase the pressure on the environment, reshaping natural ecological cycles and creating novel interphases and interactions (state), which can facilitate spillovers through the infect-shed-spill-spread cascade (impact).

Structured approaches, such as the Driver-Pressure-State-Impact-Response framework (DPSIR), already employed by the Convention on Biological Diversity, facilitate the understanding and design of relevant and well-adapted strategies and indicators underpinned by a shared goal (Baker et al. 2022), providing a conceptual approach for the identification of sources, their effects and management options. We argue that the DPSIR is an ideal foundational framework to operationalize environmental solutions to curb disease risk at the source (Fig. 1). The objective of the DPSIR framework is to describe and contextualize the interconnections between environmental issues and the socio-economic context in which they occur (European Environment Agency 1999).

Under the post-2020 global biodiversity framework, there are several proposed indicators (Convention on Biological Diversity 2022) which might provide an auspicious foundational framework for defining a shared One Health monitoring strategy. We believe that some of these indicators can already contribute to the One Health Joint Plan of Action’s activity 2.1.4 “*Develop a One Health indicator framework to monitor the health of humans, wildlife, domestic animals, vectors and the environment*…” (Table 1). For example, a reduction in the rate of land-use change is envisaged as part of the post-2020 Biodiversity Framework, with a target of protecting at least 30% of land and sea by 2030. This is also included in the UN 2030 Agenda under Goal 15, with indicator 15.3.1 measuring the “*Proportion of land that is degraded over total land area*” and is part of the One Health Joint Plan of Action with activity 6.1.8 “*Convene relevant sectors to facilitate integrated land- and sea-use planning…”*. This indicator is also directly relevant to monitor NHID risk as land-use change modifies ecological interactions and cross-species transmission rates (Murray and Daszak 2013; Gibb et al. 2020; Plowright et al. 2021). Land-use change is one of the main threats to biodiversity and currently the primary source of pandemics, causing the emergence of more than 30% of novel diseases since 1990 (Newbold et al. 2016; IPBES 2020). The effects of land use change shape the environment and induce impacts at different scales, driven by population pressure (e.g. urbanization), demand for goods (e.g. deforestation), and agricultural expansion (e.g. food), and it is often the result of complex socio-economic and biophysical processes (IPBES 2020; Plowright et al. 2021).

**Table 1:** Table of proposed headline indicators of the Convention on Biological Diversity which are potentially relevant for representing mechanisms by which environmental disruption facilitates the emergence of zoonotic infectious diseases.

Indicator	Goal, Target	Mechanism	Rationale	Reference
A.1 Red list index of ecosystems A.2 Extent of natural ecosystems	Goal A, Target 1	Ecosystem degradation/restoration	Ecosystem degradation from land-use change is one of the main drivers of zoonotic disease emergence risk. Habitat degradation acts as a top-down pressure, with effects at multiple levels altering community structures, leading to changes in infection, shedding, and transmission patterns. Ecosystem degradation increases risk of livestock–wildlife and human–wildlife contact that might lead to spillover events. Change in species' suitable habitat (from degradation/restoration processes) affects the ecological cycles of several disease systems by altering host species co-occurrence, their density, their connectivity, etc	(Gibb et al. 2020; IPBES 2020; Morand and Lajaunie 2021b)
A.3 Red list index A.4 The proportion of populations within species with an effective population size > 500	Goal A, Target 4	Biodiversity loss	The decline of biodiversity alters the composition of biological communities and their pathogen transmission dynamics. Loss of species from an ecosystem might reduce the dilution effect provided by the presence of several non-competent pathogen hosts	(Ostfeld and Keesing 2000)
1.1 Per cent of land and seas covered by biodiversity-inclusive spatial plans	Target 1	Land-use change	The protection of biodiversity and the prevention of ecosystem degradation via land-use mechanisms reduce the risk of emerging infectious diseases. Natural ecosystems can regulate the risk of pathogen transmission via dilution effects and bear lower risk of livestock–wildlife and human–wildlife contact that might lead to spillover events	(Murray and Daszak 2013; Gibb et al. 2020; IPBES 2020)
2.2 Area under restoration	Target 2	Ecosystem degradation/restoration	Ecosystem restoration, although generally positive, may lead to new ecotones which can potentially modify (including increase) transmission dynamics. This is especially relevant in ecosystems where the animal community has been depleted from habitat loss, and new individuals are secondarily re-colonizing the system	(Keesing et al. 2010; Morand and Lajaunie 2021b; Plowright et al. 2021)
3.1 Coverage of Protected areas and OECMS	Target 3	Ecosystem degradation/restoration	The protection of biodiversity and the prevention of ecosystem degradation via land-use mechanisms reduce the risk of emerging infectious diseases. Natural ecosystems can regulate the risk of pathogen transmission via dilution effects and bear lower risk of livestock–wildlife and human–wildlife contact that might lead to spillover events	(Keesing et al. 2010; Terraube and Fernández-Llamazares 2020; Plowright et al. 2021)
5.1 Proportion of fish stocks within biologically sustainable levels	Target 5	Direct use of wildlife	Fish-borne diseases include mainly bacterial and parasitic zoonotic diseases. Just like with intensified agriculture, fish farms also have top-down environmental effects, affecting the normal disease dynamics in nature and altering ecosystems	(IPBES 2020; Kock and Caceres-Escobar 2022)
6.1 Rate of invasive alien species establishment	Target 6	Ecosystem degradation (land use change, deforestation, urbanization)- Biodiversity loss/- gain	Invasive alien species can have direct and indirect effects on species and whole ecosystems, they can alter the composition of wildlife hosts and can bring novel pathogens into a system. Therefore, keeping track on them can be a good proxy of ecosystem degradation and resulting changes in normal eco-epidemiological dynamics	(Chinchio et al. 2020; IPBES 2020; Kock and Caceres-Escobar 2022)
9.1 Benefits from the sustainable use of wild species	Target 9	Direct use of wildlife	Wildlife use and trade is considering a direct threat to biodiversity and an interface of risk for health (especially live animal markets). Ensuring sustainable, legal, and safe use and trade is a key element of prevention	(IPBES 2020; Kock and Caceres-Escobar 2022)
10.1 Proportion of agricultural area under productive and sustainable agriculture	Target 10	Ecosystem degradation/restoration	Agricultural areas are often a source of human–wildlife or livestock–wildlife contact. Sustainable agricultural practices can reduce disease emergence risk by separating areas of wildlife presence from areas of human activities (land sparing), but can also increase spillover risk where areas serve both for production activities (especially livestock) and as wildlife habitat (land sharing)	(Murray and Daszak 2013; Gibb et al. 2020; IPBES 2020; Plowright et al. 2021)
10.2 Progress towards sustainable forest management	Target 10	Ecosystem degradation/restoration	Both deforestation and reforestation modify, directly or indirectly, current habitats, ecotones, and interspecific contact rates. Reducing deforestation is paramount to reducing spillover risk from human–livestock–wildlife contact, and for maintaining the natural dynamics of pathogen transmission within ecosystems	(Murray and Daszak 2013; Gibb et al. 2020; IPBES 2020; Plowright et al. 2021)
12.1 Average share of the built-up area of cities that is green/blue space for public use for all	Target 12	Ecological restoration	Ecological restoration modifies pathogen dynamics, creating new ecotones which might increase disease emergence risk	(Speldewinde et al. 2015; IPBES 2020)
15.1 Number of companies reporting on disclosures of risks, dependencies and impacts on biodiversity	Target 15	Ecosystem/restoration Biodiversity loss/gain Direct use of wildlife	Keeping track on the impacts on biodiversity (direct and indirect) can be used to assess the state and health of ecosystems, and the associated risk of pathogen emergence	(IPBES 2020)

The first two columns report the indicator name and associates goal(s) and target(s), following the Kunming-Montreal Global Biodiversity Framework (Convention on Biological Diversity 2022); in the remaining columns we propose the mechanisms and rationale by which each indicator can be connected to zoonotic disease risk, mentioning relevant literature references

Reducing global deforestation rates is also part of the United Nations Strategic Plan for Forests 2030 and 2030 Agenda for Sustainable Development (Goal 15). Forest areas are rich in wildlife diversity and zoonotic pathogens, making deforestation highly correlated with disease emergence risk (Morand and Lajaunie 2021b), especially if coupled with livestock expansion which acts as an amplifier of risk (Rohr et al. 2019; IPBES 2020; Morand 2020; Kock and Caceres-Escobar 2022).

Importantly, not all environmental interventions will necessarily decrease disease risks. In the UN decade of restoration, environmental interventions cannot be disconnected from disease risk analysis. For example, reforestation efforts could increase suitable habitat for vectors or hosts—such as ticks, mosquitoes, or rodents—creating novel ecotones and potential threats (Speldewinde et al. 2015; Morand and Lajaunie 2021b). Therefore, new paradigms need to recognize the potential undesired effects of ecological restoration and implement surveillance systems in accordance with threats. Complex socio-environmental systems require understanding all elements shaping human, animal and environmental health risks (Di Marco et al. 2020; IPBES 2020). Defining and co-constructing interventions with local communities is critical to ensure relevance, acceptability, impact, and long-term commitments (Peyre and Goutard 2022).

## Pandemic Prevention and Future Policies

The COVID-19 pandemic showed, once again, the global vulnerability of social and health systems to NHID (Johnson et al. 2022). Despite rapid global investment in post-COVID recovery initiatives, it is unlikely current health systems can respond timely and adequately to other similar crises, especially if multiple pathogens emerge at the same time. These systemic vulnerabilities have been recognized and emphasized before (Morse et al. 2012; Machalaba et al. 2018), and it is now clear that operationalizing pandemic prevention should receive as much attention as other elements of an integrated One Health response. This operationalization should learn from existing initiatives to tackle other global crises, such as the strategic plan of the Convention on Biological Diversity or the Intergovernmental Panel on Climate Change. But who should be responsible for driving this process and how?

In the short term, we argue that existing global initiatives, such as the Global Health Security Agenda, could adopt a strategic approach to develop shared goals, targets that can then drive on-ground interventions. The DPSIR framework can serve as a foundational approach to develop targets and indicators that are relevant for pandemic prevention through environmental solutions. For example, Target 3.d of the UN 2030 Agenda aims at “*Strengthen the capacity of all countries, in particular developing countries, for early warning, risk reduction and management of national and global health risks*”. Indicators of NHID threats can be included in this target, under the overall objective of reducing the impact of disease emergencies through preventive measures aimed at preparedness, early detection, and control. Engaging the operational actors and decision-makers both at the local and national level in the early stages in defining the environmental interventions that would be needed will be a critical element to ensure their relevance and acceptability — to guaranteeing their implementation— and therefore their sustainability and impact (Goutard et al. 2015; Schulz et al. 2016; Delabouglise et al. 2017).

In the medium and long terms, we support the call and ongoing negotiations for a Pandemic Accord (World Health Organisation 2023), to guide and articulate interventions to strengthen all four phases of the integrated One Health response. Such treaty should be evidence-based, transformative, equitable, and in accordance with other relevant conventions, especially multilateral environmental agreements aimed at climate change and global biodiversity conservation (Johnson et al. 2022; Phelan and Carlson 2022). We argue that such treaty should also incorporate existing environmental targets and indicators that are relevant to NHID sources (e.g. the reduction of global deforestation and land-use change rates) and develop dedicated targets that are otherwise not represented in multilateral environmental and health agreements (e.g. the reduction of human activities in known hotspots of disease emergence risk). The One Health Joint Plan of Action can provide the overarching operative framework, and as a first step the Quadripartite could facilitate a global consultation for defining specific targets of zoonotic NHID reduction, evaluate the use of existing environmental indicators (Table 1), and discuss the need for new indicators. Existing working groups, such as One Health High Level Expert Panel (OHHLEP, https://www.who.int/groups/one-health-high-level-expert-panel), can serve as scientific bodies to instruct a global consultation process for the definition of pandemic risk reduction goals, targets and indicators under a DPSIR framework. Such framework could then be undertaken by individual countries, as well as large coalition initiatives such as PREZODE (https://prezode.org), which aims to prevent pandemic risks by promoting a change in paradigm towards prevention and early control with a bottom-up approach.

Financial mechanisms to support the operationalization of an integrated One Health response should be transectoral and international, building on work already in place by initiatives such as the Global Preparedness Monitoring Board (https://www.gpmb.org), or the High-Level Independent Panel on Financing the Global Commons for Pandemic Preparedness and Response by the G20 (HLIP, https://pandemic-financing.org). Existing financial frameworks from climate change (i.e. carbon financing), such as REDD + , can help provide the methodological basis and guidance for expanding and developing targeted financial mechanisms to operationalize One Health globally. In this sense, the recently established Financial Intermediary Fund for Pandemic Prevention, Preparedness, and Response (World Bank 2022) is a promising avenue to achieve a truly integrated One Health strategy, but it requires clear mechanisms for supporting the definition of national-scale prevention strategies that address the environmental component of zoonotic disease risk. Health benefits resulting from environmental protection should also be explicitly included in environmental financial mechanisms, such as the Global Environment Facility and the Green Climate Fund. These initiatives should deliver equal attention to all elements of One Health, from prevention to recovery. Threats to the environment are unavoidable in a world where socio-economic development is still largely linked to increased use of natural resources, but decreasing the likelihood that environmental pressure leads to NHID is an attainable goal that current policies should strive for.
